# Validity and inter-rater reliability of ankle motion observed during a single leg squat

**DOI:** 10.7717/peerj.12990

**Published:** 2022-02-15

**Authors:** Paloma Guillén-Rogel, Cristina San Emeterio, Pedro J. Marín

**Affiliations:** 1 Institute of Biomedicine (IBIOMED), León University, León, Spain; 2 Faculty of Health Sciences, Miguel de Cervantes European University, Valladolid, Spain; 3 CYMO Research Institute, Valladolid, Spain

**Keywords:** Navicular drop, Navicular motion, Foot kinematics, Pronation, Visual assessment, Medial longitudinal arch

## Abstract

**Background:**

The single leg squat (SLS) test is a clinical functional test commonly used to evaluate clinically aberrant movement patterns of the knee. The SLS could be an interesting option to analyze ankle control in the frontal plane during dynamic load analysis. However, to date, there are no studies that have analyzed the associations between the increased subtalar joint pronation by navicular drop (ND) test and ankle control with single leg squat (SLS_ankle_) using a three-point scale. The purpose of this study was to evaluate the reliability of a clinical observation method to assess and determine the relationship between navicular drop (ND) and ankle control on the SLS_ankle_ score.

**Methods:**

A total of fifty-five healthy, physically active (31 females and 24 males) volunteers participated in this study. The degree of subtalar pronation was assessed through the ND test, and the ankle control was defined as the ankle displacement in the frontal plane during the SLS.

**Results:**

We found good intra-rater and inter-rater agreement during SLS_ankle_, with Kappa values from 0.731 to 0.750. The relationship between the SLS_ankle_ and ND was significant ; the Spearman’s rank correlation coefficient was 0.504 (*p* < 0.05).

**Conclusions:**

The SLS_ankle_ score supplied the clinical practice with a reliable and valid alternative for quantifying foot mobility in comparison to the ND test.

## Introduction

The single leg squat (SLS) test is a clinical functional test commonly used to evaluate movement patterns of the lower limbs to assist clinicians with screening and diagnosis (Weeks, Carty & Horan, 2012). Visual observation movement screening tests offer an inexpensive, readily accessible, and easily applied assessment of the movement system in a clinical setting.

The SLS test is a tool to assess the risk of lower extremity injury (Ugalde et al., 2015), such as anterior cruciate ligament (ACL) injury (Yamazaki et al., 2010; Yokoyama et al., 2021), patellofemoral pain (Herrington, 2014; Gwynne & Curran, 2018), and non-arthritic hip pain (McGovern et al., 2020).

The movement patterns are used with visual rating scales (Harris-Hayes et al., 2014). The observer assesses the degree of medial–lateral knee motion during a single limb squat. Often, medial knee motion during the squat is indicative of hip abductor and/or external rotation muscle dysfunction (Ageberg et al., 2010; Crossley et al., 2011). Foot and ankle movement and mechanics, along with the hip musculature, may also have an impact on the kinematics of the lower extremity.

During closed-chain activities, restricted ankle dorsiflexion (DF) range of motion (ROM) is often accompanied by decreased sagittal plane motion of the knee, hip, and trunk, as well as increased frontal plane motion of the lower extremity (Bell, Padua & Clark, 2008). For example, during a squat, restricted DF ROM may result in excessive subtalar joint pronation and midtarsal dorsiflexion (Fong et al., 2011) tibial and femoral internal rotation, medial knee displacement, knee valgus (Macrum et al., 2012; Dill et al., 2014) and pelvis drop (Wilczyński, Zorena & Ślęzak, 2020). Decreased DF ROM was also associated with reduced quadriceps activation and increased soleus activity during the descent portion of a squat (Macrum et al., 2012). Thus, the ankle is important for evaluation during the single leg squat and plays as it has a stabilizing performance during the closed chain task (Warner et al., 2019).

The navicular drop (ND) test described by Brody (1982) is a clinical test used to evaluate rearfoot and midfoot pronation and assess the function of the medial longitudinal arch. The integrity of the medial longitudinal arch (MLA) is an important factor in kinematics and function of the lower extremities during weight bearing (Nilsson et al., 2012).

ND is measured by recording the difference (in millimeters) between navicular tuberosity height in standing weight bearing and resting standing foot position (Shrader et al., 2005). Firstly, the subject was the sitting position with both knees in 90° flexion, with the foot on the floor and then the navicular tuberosity was palpated and market. Clinical measures the distance from the navicular tuberosity to the floor. Secondly, the participant standing with weight equally distributed on both feet, clinician measures distance from the navicular tuberosity to the floor (Brody, 1982; Mulligan & Cook, 2013; Elataar et al., 2020; Allam et al., 2021). 

The navicular drop test demonstrates excellent reliability, with intra-rater and inter-rater interclass correlation coefficient values ranging from 0.914 to 0.945 (Spörndly-Nees et al., 2011; Zuil-Escobar et al., 2018). An ND ≥ 10 mm is considered an excessive amount of foot pronation (Headlee et al., 2008). Furthermore, excessive pronation of the foot has been associated with increased risk of lower extremity injuries in military cadets (Levy et al., 2006) and athletes (Michelson, Durant & McFarland, 2002).

In contrast, dynamic weight-bearing task analysis is very important to reproduce activities of daily living. The SLS could be an interesting option to analyze ankle control in the frontal plane during dynamic load analysis. However, to date, there are no studies that have analyzed the associations between the increased subtalar joint pronation by ND test and ankle control with single leg squat (SLS_ankle_) using a three-point scale.

Therefore, the aims of this study were to (1) evaluate the reliability of a clinical observation method of assessment, and (2) determine the relation between the assessment of ankle control during SLS_ankle_ and the navicular test. We hypothesized that a higher ND score would correlate with the lateral malleolus displacement during the SLS.

## Materials & Methods

An a priori power analysis was conducted to estimate the sample size. G*Power software (G*Power 3.1.9.6 Kiel University, Kiel, Germany) (Faul et al., 2007) estimated a sample size of 34 subjects (significance level = 0.05; required power = 0.80; correlation among repeated measures = 0.30). A pilot study with 6 subject was used to estimate the sample size.

### Study design

An observational study was performed between April and June 2019. The study was conducted according to the guidelines of the Declaration of Helsinki and approved by the CyMO Research Institute (Valladolid, Spain: 1.200.553). All the participants read and signed an approved, written informed consent document before data collection.

### Participants

Overall, fifty-five healthy, physically active adult volunteers, 31 females (21.3 ± 5.7 yrs., 163.5 ± 7.4 cm, 59.7  ± 7.7 kg) and 24 males (27.4 ± 12.7 yrs., 177.6  ± 7.9 cm, 76.8 ± 10.3 kg), were recruited for this study. All participants were healthy, reporting no injuries. Participants were excluded if they had any joint pathology in the hip, knee, or ankle that caused pain or restricted movement, neuromuscular disease, recent heel or knee pain, or a history of recent lower extremity trauma or elective surgery in the last six months.

### Procedures & measurements

Participants completed three laboratory sessions in this study (one familiarization session and two test sessions) at one-week intervals. All sessions were performed at the same time of day to minimize the effect of circadian rhythms. All participants were instructed to refrain from exercising for 48 h prior to testing to reduce the potential influence of post-exercise muscle soreness or fatigue on performance in the SLS_ankle_ test. During the testing session, participants carried out the following tests in a randomized order: the ND test and the SLS_ankle_ test.

### Navicular drop

Each subject was asked to stand barefoot, with weight distributed evenly over each foot. The navicular tuberosity was palpated and marked with a washable marker. With the subtalar joint in the neutral position, the distance between the navicular tuberosity and the floor was measured, in millimeters, with a caliper (Mulligan & Cook, 2013; Okamura et al., 2021).

The procedure was repeated three times for each participant. One measurement is subtracted from the other. In the cases in which this difference, expressed in millimeters, is ≥10 mm, the ND signifies an excessive pronation of the foot (Brody, 1982; Cote et al., 2005).

### Single leg squat

The SLS was evaluated with the Leg MOtion® system (Leg Motion®, Check your Motion, Albacete, Spain) in a weight-bearing position. The Leg Motion® system (Check your Motion®, Albacete, Spain) is a valid portable, and easy to use alternative to the weight-bearing lunge test to assess ankle dorsiflexion ROM in healthy participants (Calatayud et al., 2015; Romero Morales et al., 2017; Moreno-Pérez et al., 2020). A digital camera (FDR-AX33, Sony, Tokyo, Japan) registered, through video recording, the lateral displacement of the ankle. The camera was placed on a tripod 3 m in front of the participant, at a height of approximately 0.9 m from the ground. This height was aligned approximately to the level of the participants’ pelvis. Video recording were made at 50 frames per second at a resolution of 1,920 × 1,080 pixels.

**Figure 1:**
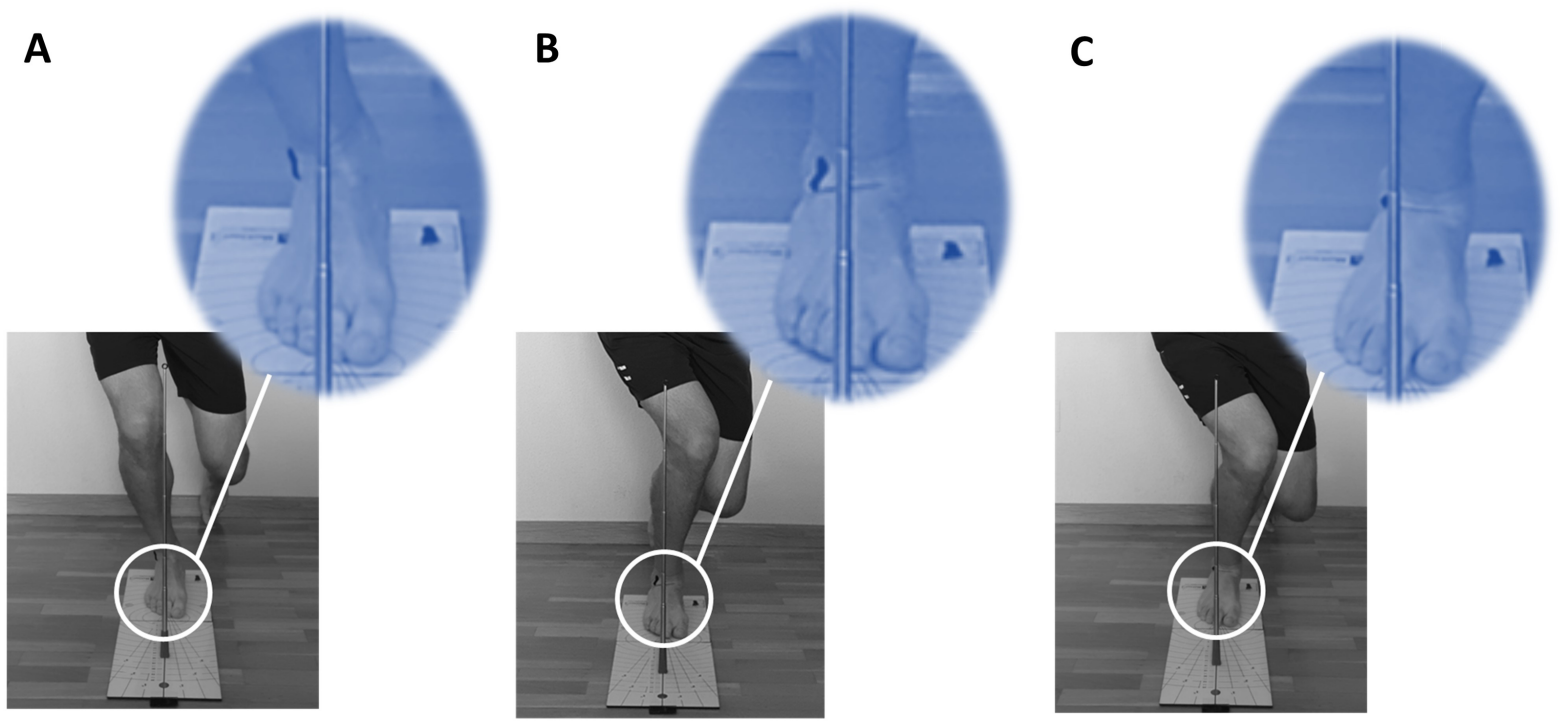
SLS_ankle_ score. (A) “0” point; (B) “1” point; (C) “2” points.

Participants stood barefoot with their feet shoulder-width apart, hips and knees extended, toes facing forward, and equal weight on both feet, and a marking strip made of masking tape (a rectangle with measures 30 × 10 millimeters) were applied to the skin over the lateral malleolus (Fig. 1).

Participants then placed one foot on the Leg MOtion platform with the second toe close to a corresponding starting line. Frontal plane ankle control was evaluated by visual observation (Junge et al., 2012) with a metal stick. A metal stick was placed along the line of the 2nd toe to indicate movement in the frontal plane during the SLS_ankle_. Ankle control was defined as the ankle displacement in the frontal plane during the SLS_ankle_.

Participants performed a SLS as far down as comfortably possible in four seconds (Nakagawa et al., 2012), keeping their trunk upright, their arms out to the side, and flexing their knee to at least 60° (Wyndow et al., 2016; Guillén-Rogel et al., 2021). In a previous study there are a consensus about the depth of the squat that a must be performed to at least 60° of knee flexion to be clinically rated as good (Crossley et al., 2011). Adequate knee flexion was visually confirmed by a researcher (Schmidt, Harris-Hayes & Salsich, 2019). Prior to testing, a researcher provided a visual demonstration of the test. Participants performed 10 practice trials with each limb to become comfortable with the task. After a 3-minute rest, each participant performed five repetitions of the SLS_ankle_ test with each lower extremity, which was videotaped.

After a 15-day wash-out period (Streiner & Norman, 2008), two examiners (a physiotherapist and an athletic trainer) were sent the video recordings to assess the motion and rate the degree of ankle control. The examiners were trained to observe each video no more than two times without any pausing or slow motion, and each had more than 10 years of video-analysis experience. The sequence of the recording was randomized with a web-based research randomizer to minimize bias (Urbaniak & Plous, 2007).

Ankle control was scored using a three-point scale (0—good ankle control, 1—reduced and 2—poor) based on the distance from the metal stick to the lateral malleolus during the SLS movement (Fig. 1). A score of 0 was recorded when raters observed that the distance between the lateral malleolus and the metal stick was unchanged from the single leg standing to squatting position. A score of 1 was given when the raters observed that the distance from the lateral malleolus to the metal stick decreased from the single leg standing to squat position. A score of 2 was recorded when the marker on the lateral malleolus was aligned with the metal stick. The subjects were rated by their poorest test performance among the five trials.

### Statistical analysis

Cohen’s kappa test was used to determine the intra-rater and inter-rater reliabilities. The kappa values were defined as poor if kappa was 0.20, fair for values of 0.21 to 0.40, moderate for 0.41 to 0.60, good for 0.61 to 0.80, and very good for 0.81 to 1.00 (Ashby, 1991).

One-way analysis of variance (ANOVA) was used to compare the ND test scores among the ankle control groups (good, reduced, or poor).

Spearman’s rank correlation coefficient was used to determine the correlation between the subjective assessment of ankle control with the scale of “good”, “reduced”, or “poor” and the ND test. All statistical analyses were conducted using SPSS (Version 22.0, IBM, Armonk, NY, USA). Effect sizes (*d*) were analyzed to determine the magnitude of an effect independent of sample size (the difference between the means divided by the pooled SD). A score of 0.5 and below was considered a *low d*, 0.51–0.8 considered a *medium d*, and 0.81 and above a *large d* (Cohen, 2013). Statistical significance was established at *p* < 0.05.

## Results

### Intra-rater reliability for the ankle control assessment

We found good agreement between the first and second test during SLS_ankle_, with kappa values of 0.750 for the right side and 0.731 for the left side.

### Inter-rater reliability for the ankle control assessment

The kappa values for the agreement between raters were 0.744 for the right side and 0.732 for the left side.

**Figure 2:**
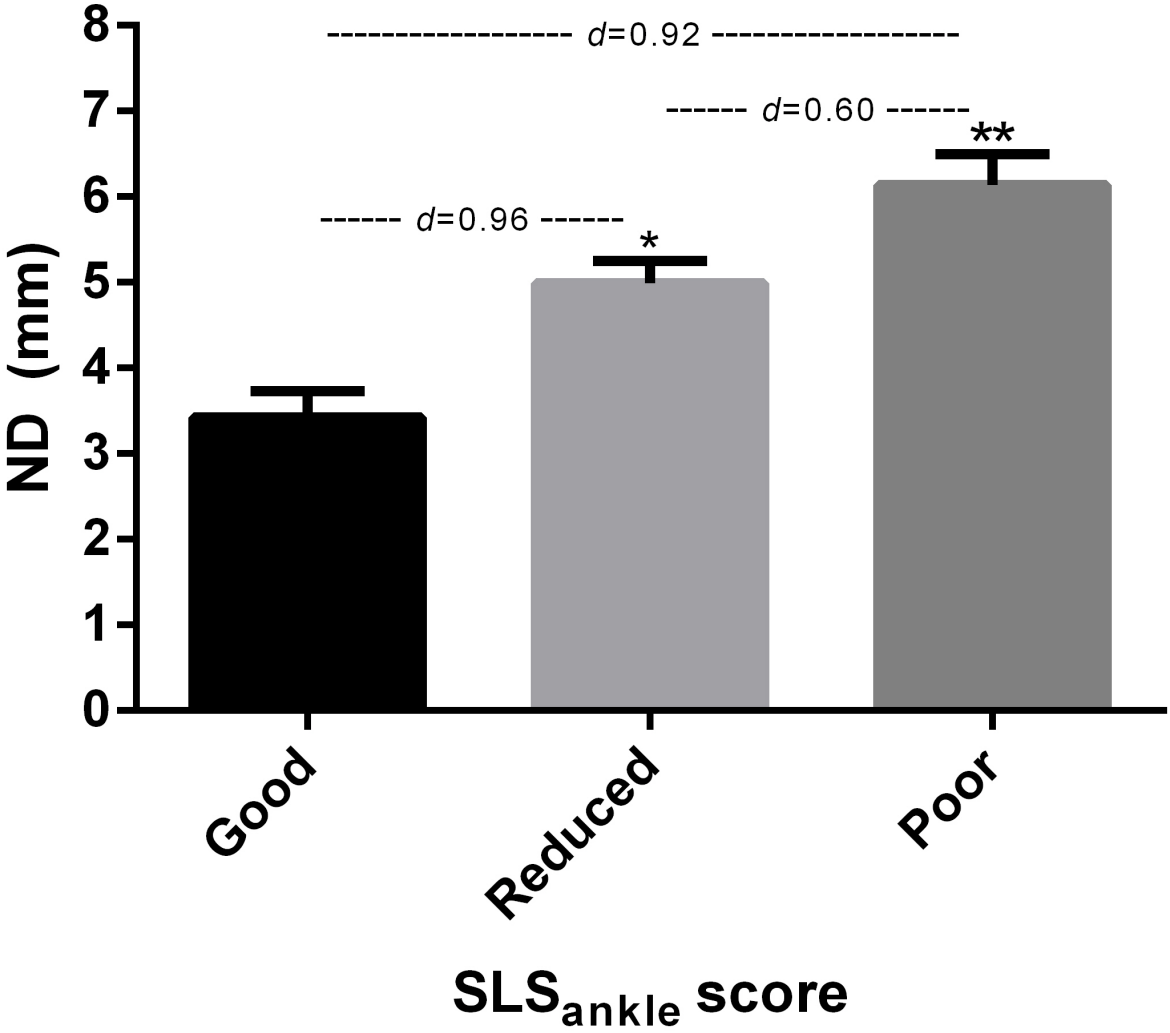
SLS_ankle_ and ND test. *Significantly different to good and poor SLS_ankle_ score (*p* < 0.05). **Significantly different to good and reduced SLS_ankle_ score (*p* < 0.05).

ANOVA showed significant differences (*p* < 0.05) for the ND test among the all SLS_ankle_ scores (Fig. 2).

Additionally, the relationship between the ankle control during SLS_ankle_ and ND tests were investigated using the Spearman’s rho correlation. The Spearman’s rank correlation coefficient was 0.504 (*p* < 0.05).

## Discussion

The aim of this study was to evaluate the reliability of a clinical observation method to assess and determine the relationship between navicular drop (ND) and ankle control on the single leg squat ankle score (SLS_ankle_). We found good intra-rater and inter-rater agreement during SLS_ankle_. The results determined that a higher ND score was correlated with lateral malleolus displacement during the SLS.

This study compared the reliability of a physiotherapy rater and athletic trainer rater; therefore, the experience level of these examiners is more likely to be an indicator of reliability (Weeks, Carty & Horan, 2012). Nevertheless, Tate et al. (2015) indicate excellent expert and novice test–retest reliability in measuring the frontal plane knee alignment during SLS.

Two-dimensional measurements of a lower extremity during a SLS, such as the frontal plane projection angle and visual evaluation, is suggested to be more cost effective and can easily be conducted in clinical settings as an alternative to three-dimensional motion capture (Hansen, Lundgaard-Nielsen & Henriksen, 2021). We found good intra- and inter-rater agreement for SLS_ankle_ score. Similarly, Stensrud et al. (2011) conducted an assessment using a two-dimensional video analysis during SLS in healthy participants and established excellent inter-rater reliability.

Various scoring systems have been used to assess dynamic alignment in the literature. Ressman, Grooten & Rasmussen Barr (2019) found that the analysis scales with a ≤ three-point rating scale show a higher inter-rater reliability compared with ≥ four-point rating scales of visual assessment of movement in the SLS test. However, there are no previous studies that have analyzed ankle control during a SLS using a three-point scale. The SLS_ankle_ score shows the visual assessment scores of good, reduced, and poor on a three-point scale. However, Perrott et al. (2012) conducted analysis of foot alignment with a two-point scale (good and poor). The primary differences between the current study and Perrott et al. (2012) and Perrott et al. (2021) were not related to the degree of pronation.

Foot pronation was described a predictor of altered joint kinetics and injuries (Brund et al., 2017), such as medial stress syndrome (Hamstra-Wright, Bliven & Bay, 2015; Menéndez et al., 2020). In addition, the alteration of the MLA can influence the biomechanics of the lower extremities. Therefore, from an injury prevention perspective, it is important to assess the deficits in active foot stabilization during dynamic pronation (Tourillon, Gojanovic & Fourchet, 2019).

The clinical implications of the test resemble the conditions of daily life, require no expensive or advanced equipment, and the experienced examiners can conduct a reliable visual assessment of the frontal plane of the ankle during an SLS test. Therefore, the use of SLS_ankle_ score is a simple screening tool that can reduce the need for health practitioners to conduct another test of pronation.

A clear strength of the test used in this study is that it is easy to use and quickly performed, which gives it strength as a clinical test where both time and reliable evaluation are essential for diagnostics. The SLS test can allow us to simultaneously make an overall assessment of the motor control of the ankle, knee, hip, and trunk. The Leg Motion® system provides a standardized device to perform the foot position during the SLS_ankle_. On the other hand, it should be noted that it may also be valid to conduct the evaluation using the malleoli instead of the navicular bone as a landmark (Kanai et al., 2020).

There are some limitations of the current study. Only healthy individuals were included, while participants with plantar heel pain or joint pathology in the hip, knee, or ankle that caused pain were excluded. Contrastingly, despite the potential benefits of using the ND test, another limitation of the study is the ND test only capable of measuring displacement in the sagittal plane, while the movement of the navicular takes place in all three planes simultaneously (Vinicombe, Raspovic & Menz, 2001). Therefore, the evaluation of pronation movement was conducted without three-dimensional analyses; however, we aimed exclusively at assessing the reliability of the test assessments.

## Conclusions

The findings of this study reveal that ankle displacement is a reliable tool to assess a single leg squat. A poor rating on the SLS test is associated with higher pronation in the ND test.

The SLS_ankle_ score has demonstrated good inter-rater and intra-rater reliability for two examiners. Therefore, the ankle assessment should be considered during dynamic assessment and supplies clinical practice with a valid alternative to quantify foot mobility in comparison to the ND test.

## Supplemental Information

10.7717/peerj.12990/supp-1 Data S1 Between-session reliability data

## Additional Information and Declarations

### Data Availability

The following information was supplied regarding data availability: The raw measurements are available in the Supplementary File.
